# Malaria surveillance in low-transmission areas of Zambia using reactive case detection

**DOI:** 10.1186/s12936-015-0895-9

**Published:** 2015-11-19

**Authors:** David A. Larsen, Zunda Chisha, Benjamin Winters, Mercie Mwanza, Mulakwa Kamuliwo, Clara Mbwili, Moonga Hawela, Busiku Hamainza, Jacob Chirwa, Allen S. Craig, Marie-Reine Rutagwera, Chris Lungu, Tokozile Ngwenya-Kangombe, Sanford Cheelo, John M. Miller, Daniel J. Bridges, Anna M. Winters

**Affiliations:** Akros, Cresta Golfview Grounds, Great East Road, Lusaka, Zambia; Department of Public Health, Food Studies and Nutrition, Syracuse University, Syracuse, NY USA; National Malaria Control Centre, Ministry of Health, Government of the Republic of Zambia, Lusaka, Zambia; Lusaka Community District Medical Office, Ministry of Community Development Mother and Child Health, Government of the Republic of Zambia, Lusaka, Zambia; Malaria Branch and US President’s Malaria Initiative (Currently Global Immunization Division), Centers for Disease Control and Prevention, Atlanta, GA USA; PATH Malaria Control and Elimination Partnership in Africa (MACEPA), Lusaka, Zambia; University of Montana School of Public and Community Health Sciences, Missoula, MT USA

**Keywords:** Malaria surveillance, Reactive case detection, Elimination, Urban, Rural, Community health worker, DHIS2

## Abstract

**Background:**

Repeat national household surveys suggest highly variable malaria transmission and increasing coverage of high-impact malaria interventions throughout Zambia. Many areas of very low malaria transmission, especially across southern and central regions, are driving efforts towards sub-national elimination.

**Case description:**

Reactive case detection (RCD) is conducted in Southern Province and urban areas of Lusaka in connection with confirmed incident malaria cases presenting to a community health worker (CHW) or clinic and suspected of being the result of local transmission. CHWs travel to the household of the incident malaria case and screen individuals living in adjacent houses in urban Lusaka and within 140 m in Southern Province for malaria infection using a rapid diagnostic test, treating those testing positive with artemether–lumefantrine.

**Discussion:**

Reactive case detection improves access to health care and increases the capacity for the health system to identify malaria infections. The system is useful for targeting malaria interventions, and was instrumental for guiding focal indoor residual spraying in Lusaka during the 2014/2015 spray season. Variations to maximize impact of the current RCD protocol are being considered, including the use of anti-malarials with a longer lasting, post-treatment prophylaxis.

**Conclusion:**

The RCD system in Zambia is one example of a malaria elimination surveillance system which has increased access to health care within rural communities while leveraging community members to build malaria surveillance capacity.

## Background

Malaria control and eventual elimination is a top priority for the Government of the Republic of Zambia (GRZ) and key malaria partners. The Ministry of Health (MOH) and Ministry of Community Development, Mother and Child Health (MCDMCH) through the National Malaria Control Center (NMCC) and with support of partners, have scaled-up proven malaria control interventions, such as artemisinin combination therapy (ACT), rapid diagnostic tests (RDTs) for supporting quality case management, and long-lasting insecticide-treated mosquito nets (LLIN) to high population coverage [1, 2]. First-line ACT is provided free of charge at public health facilities for parasitologically confirmed malaria cases. Community access to ACT has been expanded through training community health workers (CHWs) to use a RDT and treat those found positive with artemether–lumefantrine (AL), the current first-line anti-malarial treatment [3]. However, access to care remains the most significant barrier to overall systems effectiveness of quality malaria case management [4]. In addition to LLINs and improved case management, indoor residual spraying (IRS) is being applied in a targeted manner based on disease burden and population density (Pinchoff et al. personal communication) and is being monitored for insecticide efficacy through a network of partners. The scale-up of these high-impact interventions has been linked with the steady decline in malaria burden since 2006 [2, 5].

Progress against malaria in Zambia has been measured through successive Malaria Indicator Surveys (MIS) and through the national Health Management Information System (HMIS) [2, 5]. These routine data sources have been useful to track broader trends in malaria and target control interventions. For example, in Zambia these data have shown that *Plasmodium falciparum* accounts for 98 % of malaria infections [6]. Great variability in malaria transmission has been shown, prompting the GRZ to establish three broad transmission zones to characterize its heterogeneous disease burden. Further, the GRZ outlined its goal of establishing five malaria elimination areas by 2015 in their national strategic plan. Moving towards malaria elimination, enhanced surveillance systems with specific malaria focus are needed. The potential sensitivity of any passive surveillance system is limited by the treatment-seeking behaviour of the population [7], thus, increasing the availability of service providers capable of diagnosing, treating and reporting on local malaria infections is a first step towards improving the surveillance system. Still, with passive surveillance systems asymptomatic cases are missed [8, 9]. For malaria elimination programmes, identifying asymptomatic malaria infections capable of sustaining malaria transmission [10] becomes a priority [11]. Further, documenting the absence of malaria transmission is problematic when infected individuals do not seek treatment or present symptoms.

As malaria transmission declines, it becomes more focal in nature [12] and the epidemiology changes [13]. Pockets of transmission, or ‘hot spots’, characterize low transmission areas [14], and must be identified and cleared for an area to be declared malaria-free. The location of incident malaria cases identified through a passive surveillance system may be indicative of the location of asymptomatic malaria reservoirs nearby [15]. Surveillance systems incorporating case investigations and local screening for malaria not only better document the absence of malaria transmission, but can also target elimination efforts toward hot spots or pockets of sustained transmission and potentially become an intervention in themselves.

## Case description

### Reactive case detection system

Various terms have been used in the literature to describe case investigations around incident malaria cases [16]; herein, the term reactive case detection (RCD) was used [4, 15]. RCD consists of visiting the home of an incident malaria case detected passively (i.e., at a health facility or by CHWs) and testing all members of households within 140 m of index household for residual malaria infection. At low levels of malaria transmission, malaria infections are often highly focalized, especially in the household of known infected individuals [12, 15, 17]. At community level, RCD improves surveillance by finding and treating infected individuals that may be asymptomatic or simply did not seek treatment for their symptoms. Furthermore, RCD may generally decrease the reservoir of infectious individuals thereby reducing malaria transmission.

**Fig. 1:**
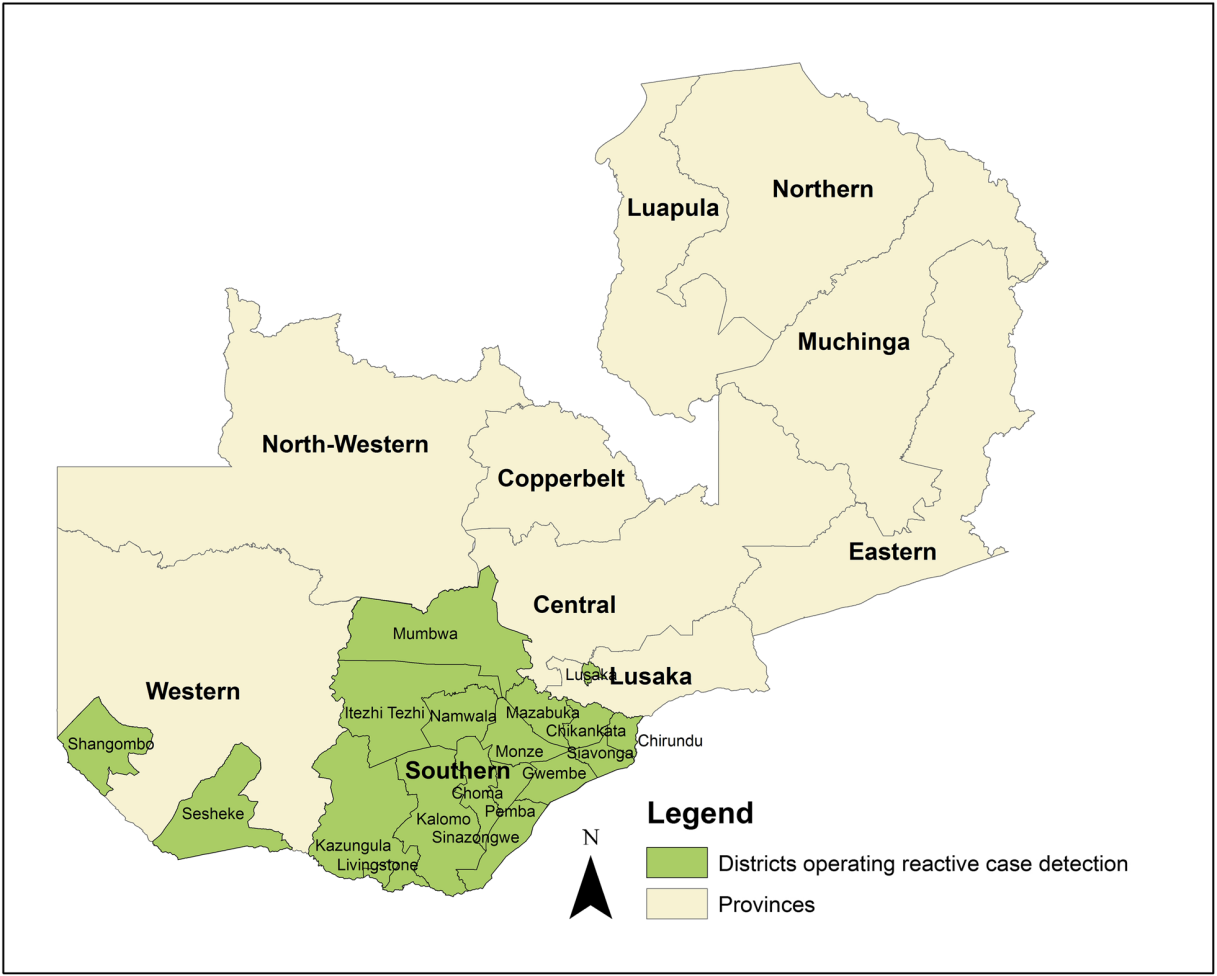
Map of areas of Zambia conducting reactive case detection

**Fig. 2:**
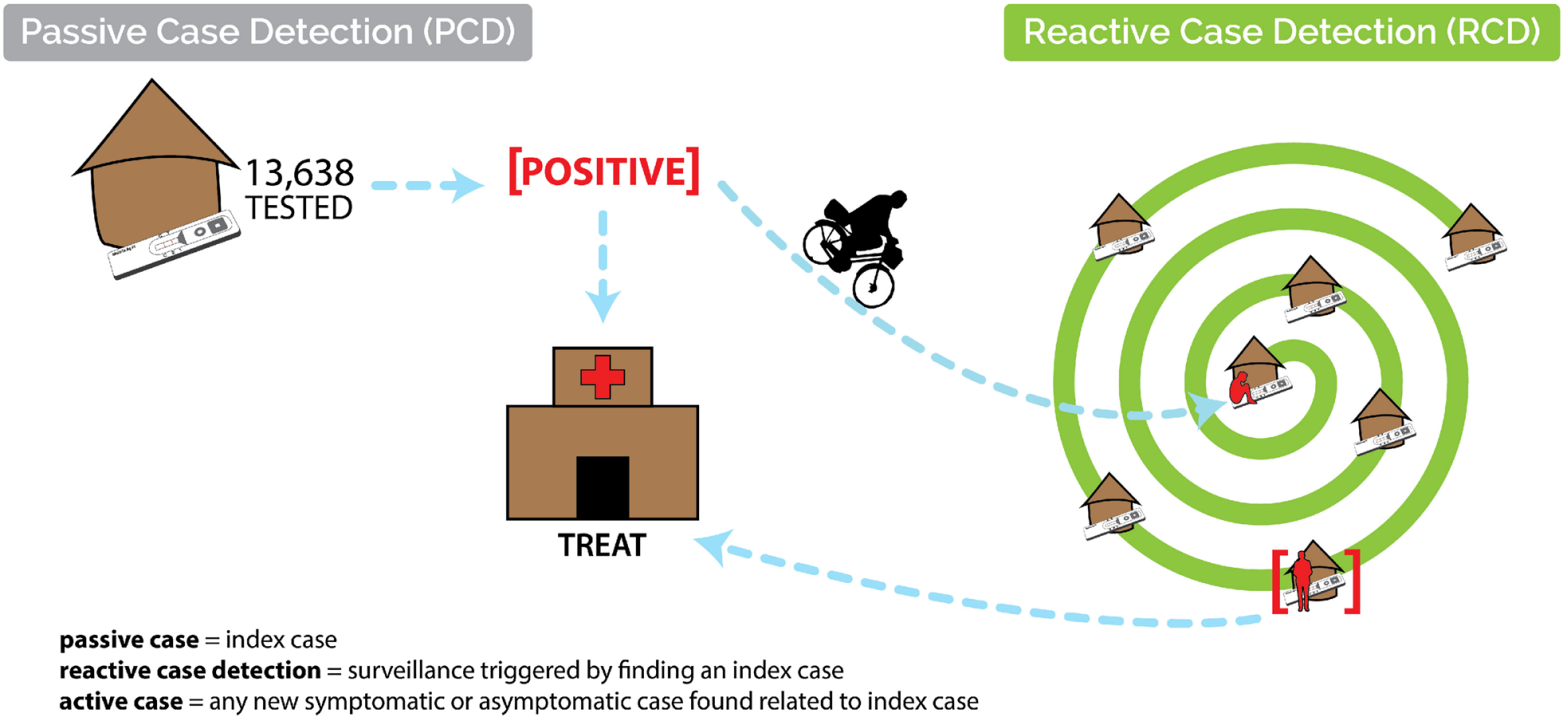
Schematic of RCD system in rural areas. A system of passive and reactive case detection includes the follow-up of confirmed malaria cases detected at clinics and health posts. Community health workers then test and treat in the house and neighbouring houses of the index case

The GRZ and partners developed an RCD system to improve malaria surveillance in response to the elimination target outlined in the 2011–2016 National Malaria Strategic Plan to establish five malaria-free areas. Zambia’s RCD programme relies on the routine health system, which tests suspected malaria cases via microscopy or RDT. In Zambia, microscopy is limited to urban areas and referral hospitals, thus, the vast majority of parasitologically-confirmed cases, especially in rural health centres and at community level, are confirmed by RDT. The sensitivity and specificity of RDTs used are checked periodically by the NMCC. In areas of Zambia conducting RCD (Fig. 1), all confirmed incident malaria cases residing within the catchment area of the health clinic or CHW post where treatment was sought are considered for a response aimed to be conducted within 1 week (Fig. 2). In urban Lusaka, due to the high population mobility and prevalence of imported malaria, RCD responses are only initiated if the incident case does not report a history of travel outside Lusaka District within the previous month. Risk of onward transmission from imported cases in Lusaka is likely low [18]. Furthermore, incident cases with a history of confirmed malaria, i.e., having a positive RDT, within the previous month are excluded from additional follow-ups in urban settings due to the possibility of HRP2 antigen persistence. Incident cases with a history of travel are not excluded from RCD in rural Southern Province because of the heightened risk of onward transmission. The key differences in the implementation of the RCD system between rural and urban contexts are outlined in Table 1.

**Table 1:** Comparison of key RCD response differences in urban and rural settings

	Urban	Rural
CHW length of training	2 days	4 days initial and 2-week clinic attachment post-training
Incident case detection	Clinic	Clinic and CHW health post
Exclusion criteria	Travel within last month	–
Team composition	Environmental health technician, nurse, 2× CHW	CHW
Incentives provided	Money per day worked	Mobile-phone airtime per monthly report, bicycle, apron, bag
RCD response	Ten houses around index, including index house	140-m radius from index house
Data collection	Paper forms/tablet computers → submission to web-based DHIS2	Paper registers → m-phone submission to web-based DHIS2

*EHT* environmental health technician

Each RCD response begins at the household of the index case, the location of which is identified by the local CHW (Fig. 2). All consenting household residents excluding the index patient are tested for malaria using RDTs according to manufacturer instructions and MOH guidelines. Any individual with a positive RDT without presentation of severe or complicated malaria is treated with the standard first line ACT following MOH guidelines unless contraindicated. As per national policy, CHWs refer (a) anyone who is found to be severely ill or (b) women who are pregnant and testing positive for malaria to the nearest clinic. The RCD response is conducted systematically beginning at the index case household and moving in a clockwise pattern from household to household.

The scope of the response in RCD operations differs between urban and rural areas due to population density and the feasibility of testing a great number of individuals (Table 1). In rural areas, CHWs screen all individuals who reside within approximately 140 m of an incident case household, which typically consists of a single compound containing an average of approximately ten people. This 140 m radius was determined through analysis of mass testing and treatment data in Southern Province [19]. Depending on CHW time and commodity availability, CHWs may extend their screening outside of the 140 m radius. In urban areas, CHWs screen individuals in the same incident case household and eight households adjacent to the incident case household, which averages approximately 33 people. Fewer than eight houses may be tested in some instances where there is no apparent adjacent house.

During each RCD response, data are collected on tablet computers (Lusaka urban) or in registers (rural) from each resident of every household visited including age, recent travel and malaria history. In urban areas, additional information is collected including household GPS coordinates and IRS history; patient-level information is recorded using open-source Open Data Kit platform and aggregate figures are then uploaded into the NMCC’s instance of District Health Information System 2.0 (DHIS2; http://www.dhis2.org). In rural areas, aggregate data are submitted by mobile phones directly to the DHIS2 system. Once in DHIS2, data can be monitored for trends at all levels of the health system, from CHW health post to province. Government personnel and partners are able to review malaria data in a near real-time manner using dashboards available within DHIS2, as well as develop additional tables, graphs and maps to guide decision-making.

### Selection and training of response teams

The composition of RCD teams in Lusaka varies from those in rural districts (Table 1). Government (clinic-based) staff form part of the RCD teams in urban Lusaka. Team members include an environmental health technician (EHT) acting as the team lead; a nurse on-hand to administer anti-malarials and to provide referral if needed; and two CHWs who are intimately familiar with their community to navigate to the household of the index case, to administer RDTs, and to maintain records. In rural areas, where available clinic staff are fewer in number and travel distances are greater, RCD is performed by a single CHW. Selection criteria for CHW involvement in RCD activities include previous CHW training and grade 12 diploma or equivalent literacy. In urban Lusaka, catchment areas are geographically smaller, with travel distances from clinic to incident malaria case typically no further than 6 km. In rural areas, catchment areas are much larger (approximately 300 km^2^) and travel distances from health post to index household for RCD can exceed 25 km.

**Fig. 3:**
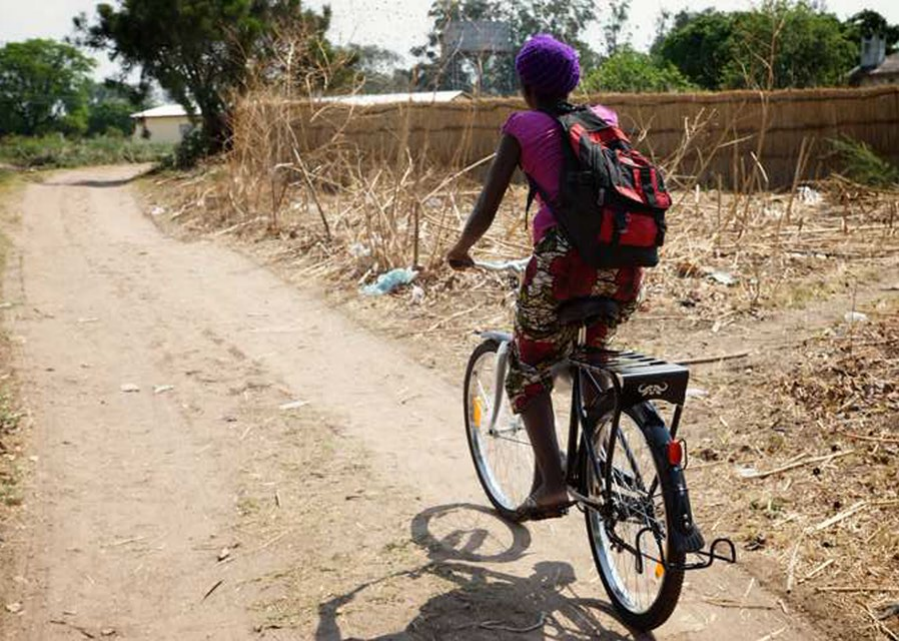
Community health workers involved in the surveillance system travel long distances to follow-up malaria incident cases within the community, or to collect commodities to test and treat for malaria. To accomplish their duties CHWs receive ‘Test for Life’ bicycles, which also serve as an incentive for their work

In rural areas, CHWs are typically “attached” to a health post (Fig. 2). This health post can vary from an official government structure to a central community location, such as a certain tree or the CHW’s own household. Where appropriate, a CHW may also visit the home of symptomatic individuals within their community. Guidelines for health post catchment area population suggest catchment areas should be limited to 500 individuals, although most health post catchment areas include a larger population from around 750 to 1000. In rural areas, RCD activities are initiated from a CHW’s health post; thus the responses are integrated with and benefit from existing CHW activities and community relations (Fig. 3).

Reactive case detection training is both classroom and field-based (2 days for urban teams, 4 days for rural CHWs; Table 1). Materials covered include background on malaria transmission in Zambia, programme objectives, in-depth review of RCD and data management protocols, and case management training for uncomplicated malaria. Standardized training to administer RDTs is included, in addition to referral protocol for severe and complicated malaria cases or other issues encountered during responses. Once training is completed, CHWs are required to complete a 2-week clinic attachment under the supervision of clinic staff to ensure quality of service provision. Follow-up, 3-day refresher trainings that emphasizes case-, data- and logistics-management are conducted each year to maintain provider and CHW skills, particularly as fewer and fewer cases of malaria are observed. Supervision of the RCD systems is provided by GRZ clinic, district and central staff and partners and consist of quarterly clinic and community visits, as well as ongoing monitoring of RCD data through the NMCC DHIS2 instance.

### Trends in malaria measured through the surveillance system in 2014 (stratified by Lusaka/Southern Province)

In 2014, the 20 health centres participating in the Lusaka District RCD system detected 3688 confirmed incident malaria cases, of which 3080 (83.5 %) reported recent travel history and were excluded from further RCD activities. Of the remaining cases, 144 underwent a full RCD response; limitations with human resources were the primary reason for not following up an eligible case. A total of 3955 individuals were tested and only 66 RDT positive individuals were found during these RCD activities, resulting in an RDT positivity of 1.94 %. The number of positive individuals found during RCD responses varied from zero to five, with 71.5 % of responses finding no malaria-infected household members or neighbors of the incident case.

Also in 2014, throughout Southern Province and other select districts, CHWs working in the RCD system tested 225,339 individuals who presented with symptoms for malaria. A total of 53,463 confirmed incident malaria cases were detected and eligible for case investigations (RDT positivity = 23.73 %) (Table 2). Subsequently, CHWs performed case investigations in 1848 homes, testing an additional 143,295 individuals regardless of symptoms, and further identifying 22,201 confirmed malaria infections (RDT positivity = 15.49 %). CHWs operate as volunteers and their ability to conduct case investigations is affected by various factors including seasonality, other responsibilities and motivation. Further research is planned to examine how these factors are affecting case investigations.

**Table 2:** Summary of RCD activities in rural areas of Southern Province, Central and Western Provinces during 2014

	RDTs administered	RDTs positive	RDT positivity (%)	Treatments given
During routine passive case detection	225,339	53,463	23.7	51,578
During reactive case detection	143,295	22,201	15.5	21,497

## Discussion

The RCD system presented herein aims to further Zambia toward malaria elimination by: (a) prompting CHWs to perform case investigations in areas affected with recent confirmed malaria, therefore expanding access to health care and (b) improving the malaria surveillance network to maximize the number of diagnosed and documented incident malaria cases to guide intervention decisions. As of July 2014, more than 1800 CHWs have been trained and are participating in RCD in Southern, Central and Western Provinces. The effectiveness of RCD at reducing malaria transmission has not been tested nor demonstrated, however current plans are underway by this consortium to test the effectiveness of RCD at reducing malaria transmission in a rural environment. Efforts are also underway to further examine the RCD intervention in urban Lusaka.

“Robust and responsive” surveillance systems have been noted as critical to achieve malaria elimination. As such, a number of malaria endemic countries have developed surveillance approaches aimed at intervening and documenting progression to Zambia; these systems exhibit both similarities and differences to Zambia’s system [20]. Many of these systems are stand-alone, closed (versus open-source) systems, compared to Zambia’s system which is open source and integrated into the central HMIS. Cambodia, for example, utilizes the Malaria Information System (MIS), a stand-alone system developed in MS Access with communication from two additional alert systems for malaria positive and resistance data [20]. This system also provides SMS alert messages to various levels of the Cambodia MOH, an aspect which the Zambia system has not included. Swaziland has made significant headway in their malaria surveillance approach. GPS-enabled tablets are used for case investigation and intervention data collection and submission of passively-detected malaria case data is made via a toll-free hotline, followed by manual entry onto a central server. These data are then communicated to surveillance agents via SMS in order to conduct case investigation and intervention. Unlike many countries, the Zambia system places primary dependence on the local CHW (voluntary) workforce for RCD. CHWs are relied upon to follow-up passively-detected cases identified at clinics, or even by CHWs during their routine community work. During case follow-ups, CHWs collect and submit data to the DHIS2 via mobile phone. The Zambia system has been developed with the CHW workforce as a primary user: simple mobile phones, clear indicators, training materials and job aides make the system understandable and approachable by CHWs. The use of the local CHW workforce as the primary drivers of the system is key to sustained surveillance for achievement and maintenance of elimination.

Declaring elimination involves the surety that local malaria transmission is not occurring. Thus, determining if prevalent cases of malaria are locally acquired is of great importance in areas such as Lusaka and Southern Province, which border regions of intense malaria transmission [21]. Ensuring that malaria transmission is not re-established will likely necessitate continued RCD for malaria cases presenting within malaria free zones throughout Zambia.

**Fig. 4:**
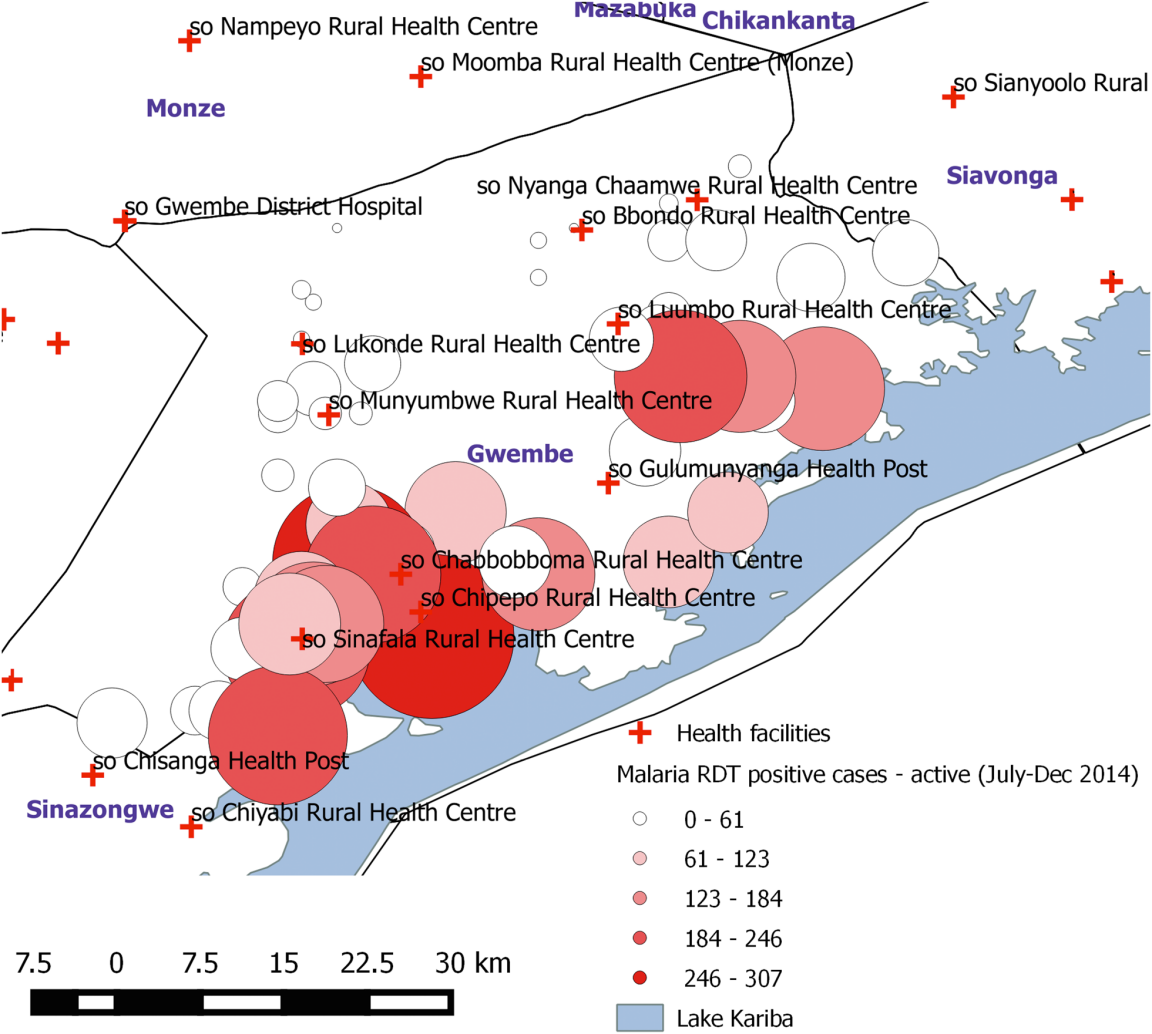
Example of mapping capacity of the RCD system. Size of the *circles* represent the number of people tested by CHWs for malaria during RCD in Gwembe District, Southern Province from July–December 2014. Suspected malaria cases tested over this time period ranged from 21 to 1703. This map taken directly out of the DHIS2 interface illustrating the usability of the system

Further, a key benefit of the RCD system is the ability to map incident malaria cases and RCD outcomes from nearby households (Fig. 4). The data-rich maps generated can guide intervention decisions, such as where to target LLIN distributions or IRS operations. The heterogeneity of malaria transmission lends itself well to targeting both vector control and chemo-elimination interventions [17], especially in elimination settings where malaria may not be the most pressing public health concern. For example, in 2014 the GRZ planned their Lusaka District indoor residual spray (IRS) campaign taking into account RCD data from current and previous years to identify persistent malaria hotspots within health facility catchment areas. Parasite prevalence identified during RCD may be a better indicator for the prevalence of locally-acquired malaria infections than incidence, and mapping test positivity from responses can indicate where foci of residual transmission remain, thus directing interventions in a targeted manner.

With the enhancement of surveillance data and the increasing heterogeneity of malaria transmission in Zambia, malaria elimination appears to be within sight for areas of Southern Zambia and Lusaka District. The Malaria Policy Advisory Committee (MPAC) recently provided guidance allowing countries to declare achievement of sub-national elimination with country-wide certification remaining dependent upon full WHO review [22]. Some countries including the Philippines and Indonesia are setting out to declare sub-national elimination one province at a time with progression towards national-level certification [23, 24]. A subnational elimination approach has been discussed within Zambia; certainly moving from an all or nothing nationwide malaria-free certification to a sub-national certification would likely encourage local communities, district health teams and Ministries of Health towards gradual attainment of the ultimate goal of national malaria elimination. Further, the definition of what constitutes a malaria-free area needs clarification for those regions bordering high transmission areas. Lusaka, for example, will likely continue to have prevalent malaria despite having eliminated local and onward transmission.

The RCD system implemented in Zambia was designed to be operational with limited continual funding. In crafting the system, key personnel at the community, district, provincial and national levels were consulted to ensure the system’s long-term sustainability. First, the system uses established personal relationships between CHWs and community members. In rural areas, CHWs are respected primary care givers making their involvement in the RCD system crucial. Second, monitoring of RCD activities is focused on a simple and concise set of indicators, especially in the rural areas. Third, the use of mobile phones for reporting in an environment where mobile phone use is pervasive builds on existing technical expertise and reduces logistical challenges associated with routine collection of paper-based reports. Finally, the use of a web-based server such as DHIS2 to host data allows for all members involved in the information chain to access near real-time feedback characterizing the malaria landscape in their constituencies. The long-term sustainability of RCD has been demonstrated in Lusaka District, where the Community District Medical Office incorporates the maintenance costs of the system in its annual budget, which includes increased numbers of RDTs and staff time. Initial RCD training and startup activities represent the major costs; once the RCD system is operational it is quite inexpensive and feasible to maintain. Relying on volunteer CHWs in rural areas may be a threat to the sustainability of the system given the potential for high CHW work burden, particularly during peak malaria transmission season. Perhaps adapting the Lusaka RCD incentive structure, where CHWs are paid per response, or development of a formal, paid community health workforce in Zambia will need to be considered. A full costing analysis of this system is forthcoming.

There are key areas where the RCD system could be improved. Firstly, the sensitivity and specificity of the malaria diagnostic test used to determine an incident malaria case will directly affect the number of responses and ultimately the efficacy of RCD. With any diagnostic test, the proportion of positives that are false increases as the true prevalence declines. For example, if the true RDT positivity is 1 % and specificity of the RDT is 95 %, five false positives would be identified for every true positive. While it is important to improve sensitivity [8] preferably to well below the detection threshold of microscopy or RDTs [25], specificity must also be addressed to ensure that resources in elimination settings are not wasted on false responses. Secondly, using a simpler drug regimen would also likely improve treatment adherence. Currently the Zambia RCD system uses AL, which is taken twice a day for 3 days. Development and approval of a once a day regimen would likely improve compliance, particularly among individuals with asymptomatic malaria infections [26].

## Conclusions

The RCD system in Zambia is one example of a RCD system applied in the malaria elimination context. This system increases the granularity of routine surveillance systems to better capture both symptomatic and asymptomatic malaria cases and may improve access to healthcare. The malaria burden is then better characterized and able to guide interventions and progress toward elimination. RCD system expansion and continuity has potential to propel Zambia further in its push towards malaria elimination by enhancing surveillance to provide a strong platform for targeting essential malaria interventions.

## Abbreviations

ACT: artemisinin combination therapy
AL: artemether–lumefantrine
CHW: community health worker
DHIS2: District Health Information System 2.0
EHT: environmental health technician
GRZ: Government of the Republic of Zambia
IRS: indoor residual spraying
LLIN: long-lasting insecticide-treated mosquito net
MOH: Ministry of Health
MIS: Malaria Indicator Survey
NMCC: National Malaria Control Centre
RCD: reactive case detection
RDT: rapid diagnostic test
